# Chronology of Alcohol Dependence: Implications in Prevention

**DOI:** 10.4103/0970-0218.42375

**Published:** 2008-10

**Authors:** Narayana Manjunatha, Sahoo Saddichha, Baxi NP Sinha, Christoday RJ Khess, Mohan K Isaac

**Affiliations:** Department of Psychiatry, NIMHANS, Bangalore, India; 1 BA MBBS DPM Partner, Division of Clinical Research, Emergency Management and Research Institute (EMRI) Hyderabad, India; 2 (Crisis Resolution and Home Treatment) Tees, Esk and Wear Valleys NHS Trust, University Hospital of North Tees, Hardwick Road, Stockton-on-Tees, Cleveland, TS19 8PE, United Kingdom; 3 Centre for Addiction Psychiatry, Central Institute of Psychiatry, Ranchi, India; 4 Department of Population Neurosciences, University of West Australia, Australia

**Keywords:** Alcohol dependence, chronology, primary prevention

## Abstract

**Background:**

Study of the chronology of criteria of dependence in alcohol dependence syndrome (ADS) can enable us design strategies for the prevention for ADS, which aims at reducing the occurrence of ADS.

**Objective:**

To study the age-wise and order-wise chronologies of ICD-10 (DCR) dependence criteria in individuals with ADS.

**Materials and Methods:**

Consecutively admitted and consenting inpatients with ICD-10 (DCR) diagnosis of ADS were evaluated in a structured interview after detoxification using Semi-Structured Assessment for the Genetics of Alcoholism (SSAGA)-II.

**Results:**

The total sample size was 81. The mean ages at the first onset of alcohol use, development of the first criterion and International Statistical Classification of Diseases and Related Health Problems 10^th^ Revision (ICD-10) dependence was 18.72 years (SD, 6.84), 24.33 years (SD, 9.21) and 27.51 years (SD, 9.28), respectively. In age-wise chronology, tolerance, loss of control and craving were present in 97.53%, 80.24% and 79%, respectively, of our study sample. In order-wise chronology, either craving (16%) or tolerance (71.6%) was present as the first criterion and the presence of craving (16%), tolerance (21%) or loss of control (18.5%) was observed in the first criterion in 55.5% of the subjects.

**Conclusions:**

Knowledge of chronology, its frequencies and time duration between various milestones in the development of the dependence criteria may enable the selection of the target population at an early stage. The pattern of development of dependence may provide us with an opportunity for interventions to reduce the incidence of ADS, as a step toward primary prevention. Adequate training of the primary care personnel and early psychiatric referral may help in the reduction in the incidence of ADS.

## Introduction

Alcohol Dependence Syndrome (ADS) gained public health importance immediately after Edward and Gross(1) described the typical behavioral clusters pertaining to ADS. The same criteria are used to diagnose ADS worldwide in both International Statistical Classification of Diseases and Related Health Problems 10^th^ Revision (ICD-10) and Diagnostic and Statistical Manual of Mental Disorders (DSM)-IV, with slight modifications. In ICD-10, the presence of minimum three of the six criteria is required to diagnose ADS,(2) and the cut-off for these three criteria is supported by studies on alcohol dependence.(3–4)

Secondary and tertiary prevention are the predominant areas of research in the current management of ADS, i.e., detoxification, relapse prevention and early diagnosis. A majority of alcohol users are generally observed in general practice settings(5) and in psychiatric clinics of general hospitals, where alcohol users are usually assessed for the criteria of dependence. If a person fulfils three or more dependence criteria of ICD-10, ADS is diagnosed and the management follows the established algorithms of secondary and tertiary prevention. However, it is quite likely that the same individual has had contact with a primary care set-up before the development of ADS, where he may have received treatment for other alcohol-related conditions such as gastritis, hepatitis and related medical disorders.(6) At this point, he may not have developed the complete syndrome of ICD-10 ADS (i.e., three or more criteria).(7) However, he may have had one or two criteria of dependence where a specialist referral may be helpful before ADS diagnosis is made. The patients in this category are called ‘diagnostic orphans’ as reported by Kaczynski and Martin;(3) other workers have adopted this categorization.(8,9) These individuals may have the high risk for developing ICD-10 dependence syndrome and eventually present with three or more criteria.(8) Moreover, diagnostic orphans are more likely to seek help for alcohol-related problems before developing complete dependence syndrome.(4) The questions here are what is strategy for the diagnostic orphans? When should intervention be applied and what strategies should be employed? What is the strategy for alcohol users who do not fulfill the dependence criteria?

Compared with the studies conducted on ADS, those conducted on alcohol diagnostic orphans are still at the initial stages,(10) although some attempts have been made recently.(3,9,11) Very little research have been carried out with regard to the chronology of the development of ADS. Ehlers *et al.*(12) conducted a community-based study on the clinical course of alcoholism; however, they did not formulate any preventive plan for ADS. A systematic study of the chronology of the criteria has not been comprehensively carried out till date which might enable us design a strategy for the prevention and lowering the incidence of ADS.

In this study, we report a systematic study on the chronology (age- and order-wise) and the prevalence of each criterion of ICD-10 ADS of inpatients, which may enable us design strategies for primary prevention of ADS and to identify the ‘at-risk’ population efficiently, especially in routine primary care practice.

## Materials and Methods

In this study, we recruited patients admitted consecutively between October 2005 and August 2006 at the Center for Addiction Psychiatry (CAP), Central Institute of Psychiatry (CIP), Ranchi, India diagnosed with ICD-10 ADS and who provided written informed consent. The study protocol was approved by the Institute's ‘Ethics committee’. The study was conducted at the CAP, CIP, Ranchi, which is a premier postgraduate training institute in Eastern India and has a large clinical service capacity of a total of 673 psychiatric inpatient beds, including a separate 30-bedded CAP. CAP treats over 500 persons every year, including more than 350 inpatients with alcohol dependence per year with nearly 100% bed occupancy. It has a wide catchment area and serves as the primary center for the people living in immediate vicinity and a tertiary referral center for the neighboring states of India as well as neighboring South Asian countries such as Nepal, Bhutan and Bangladesh. Subjects who fulfilled the criteria for other substance dependence, those with other comorbid psychiatric disorders or general medical conditions and patients with Mini-Mental Status Examination (MMSE)(13) screening scores of less than 24, indicating impaired cognition, were excluded from the study.

As a part of the clinical protocol of CAP, a Junior Resident (trainee psychiatrist), along with informants (preferably spouse), conducted a detailed examination of the ADS patients and systematically recorded the data in case record files (CRF). CRF is a detailed semi-structured proforma specially designed for assessing substance dependence and used in the outpatient department (OPD) of CIP. Diagnosis was confirmed after discussion with a senior resident or consultant (qualified psychiatrist) in the OPD, following which the decision for admission of inpatients was taken. At the time of admission, another junior resident of CAP independently clarified the history from the patient and available informants to confirm the diagnosis. The same patient again discussed with a senior resident or consultant of CAP during ward rounds for advice on the final diagnosis and management. Patients who fulfilled the study criteria were detoxified and then interviewed by using the alcohol section of the Semi-Structured Assessment for the Genetics of Alcoholism(14) (SSAGA)-II. The details of the SSAGA-II questionnaire have been reported in another study.(15)

Since this was a retrospective study, the questions were framed individually to trigger the recall by using anchor questions pertaining to personal and impersonal or important social events and defining the technical terms.(16) Relevant information about patients was also corroborated from their respective CRF completed at the time of admission. In case of discrepancy in any of the items, the matter was discussed with the patients for consensus. At the end of interview, data was transferred to ICD-10 tally sheet of the respective items in the alcohol section of SSAGA-II. Among the first age(s) of appearance of items of each criterion, we considered the earliest age of appearance of any item as the age of the first appearance of the respective criteria of dependence (ICD-10 DCR). We considered the age of development of ICD-10 dependence syndrome as the age of onset of the third consecutive criterion, with the simultaneous presence of other two criteria (among the six criteria of ICD-10).

The criteria for ICD-10 ADS(2) are (a) a strong desire or sense of compulsion to consume alcohol [CRAVING]; (b) difficulties in controlling alcohol intake behavior in terms of the onset, termination, or levels of use [LOSS OF CONTROL]; (c) physiological WITHDRAWAL state; (d) TOLERANCE; (e) progressive neglect of alternative pleasures or interests [SALIENCE]; and (f) PERSISTENT USE DESPITE OVERT PHYSICAL OR PSYCHOLOGICAL HARM. In this study, the above mentioned key words (bold, capital) for each criterion have been used in the Discussion.

## Results

The total sample size of present study was 81. All subjects were males with mean age of 35.16 years (SD, 10.20 years). The mean duration of formal education was 11.7 years (SD, 3.98 years). Of the total subjects, 49.4% (N = 40) were engaged in skilled and semi-skilled jobs, 27.2% (N = 22) were professionals, 8.6% (N = 7) were students, 8.6% (N = 7) were unemployed and 6.2% (N = 5) were not actively employed. Further, 70.4% (N = 57) subjects were married, 27.2% (N = 22) were single and 1.2% each (N = 1) was separated and divorced. The mean monthly income of the subjects was INR 8451.2 (SD, 7901.03) [approx $187.8 (SD, 175.6)]. The residence statuses of the subjects are urban, 75.3% (N = 61) and rural, 24.7% (N = 20). Family history of alcohol dependence was noted in 77.8% (N = 63) of our study samples.

The ages of onset of the first criterion and ICD-10 dependence were 24.3 years (SD, 9.2 years) and 27.5 years (SD, 9.3 years). The duration between onset of the first criterion and ICD-10 dependence was 3.2 years (SD, 3.2 years). The time-gap between onset of alcohol use and appearance of the first criterion and from onset of alcohol use to ICD-10 dependence was 5.6 years (SD, 6.2 years) and 8.78 years (SD, 6.7 years), respectively [Table 1].

**Table 1:** Notable age of onset of different chronologies in the present study

Criteria	Age of onset (years) (mean ± SD)
Age at onset of alcohol use	18.72 ± 6.84
Age at onset of the first criteria	24.33 ± 9.21
Age at onset of the second criteria	25.86 ± 9.45
Age at onset of ICD-10 dependence	27.51 ± 9.28
Duration from onset of alcohol to the first criteria	5.61± 6.2
Duration from onset of alcohol use to dependence	8.78 ± 6.7
Duration from the first criteria to dependence	3.17 ± 3.23

This study also analyzed the two types of chronology of ICD-10 dependence: the age-wise chronology (for all criteria) and the order-wise chronology (up to only the third criterion since it fulfilled the threshold criteria for the diagnosis of the ICD-10 dependence). The age-wise chronology is analyzed for a better understanding of the course and progression of the disorder, represented as ages at which patients experienced the first onset of each criterion of dependence and its frequencies [Table 2]. The order-wise chronology of each criterion is summarized as its frequencies in their order of appearance (the first, second and third criteria) at their life-time first appearance [Table 2].

**Table 2:** Results of the present study with both age-wise and order-wise chronologies

ICD-10 criteria of dependence	Present study (N = 81)								
	
	Patient who experienced the criteria		Age at which criteria was first experienced (age-wise chronology) (years) Mean ± SD	Order-wise chronology of each criterion					
				
				First		Second		Third	
						
	N	%		N	%	N	%	N	%
Craving	64	79	26.71 ± 8.0	13	16.0	13	16.0	16	19.8
Tolerance	79	97.5	25.15 ± 9.4	58	71.6	17	21.0	2	2.5
Loss of control	65	80.2	27.76 ± 9.4	3	3.7	15	18.5	22	27.2
Salience	31	38.3	29.67 ± 7.7	0		1	1.2	9	11.1
Withdrawal symptoms	75	92.6	27.04 ± 9.8	6	7.4	29	35.8	21	25.9
Persistent use despite harm	71	87.6	27.61 ± 7.7	1	1.2	6	7.4	11	13.6

## Discussion

It can be noted that, on an average, a person uses alcohol for approximately six years before developing the first criterion of dependence and then requires approximately three to four years from the appearance of the first criterion to the development of ICD-10 dependence. Thus, the duration of criteria-free (or social drinking) stage lasts for approximately six years; further, if the alcohol use continues, then ICD-10 dependence develops clinically in approximately three to four years (or predependent stage). These ‘alcoholic diagnostic orphans’(3,4,8,9) are the best targets for preventing the development of ADS(17) since these groups are relatively more motivated for intervention at this stage,(4) and they can be targeted individually by a clinician.

In our study, we have discussed both age-wise and order-wise chronologies. However, age-wise chronology of each criterion has important limitations. Firstly, every patient does not experience each criterion of dependence, which is evident in the present study as well as in other studies.(4,8,9,11,12) Secondly, the age of onset of dependence may not be the appearance of any criteria of dependence.(12) The order-wise chronology of each criterion is very important in overcoming the limitations of the age-wise chronology. Table 1 presents the ages of the life-time first appearance of the first, second and third criteria as a whole and the time duration between important milestones, which are important in formulating a prevention plan for ADS. This can enable a better understanding of individual criterion in alcoholic diagnostic orphans for primary prevention of ADS.

*What are the criteria to be enquired in pre-dependence stage* [alcohol diagnostic orphans]? This can be answered precisely by analyzing the data of both age-wise and order-wise chronologies. In order-wise chronology, either craving or tolerance was present in 87.6% as the first criterion and the presence of craving (16%), tolerance (21%) or loss of control (18.5%) was observed as the second criterion in 55.5% of the subjects. Moreover, in age-wise chronology, tolerance, loss of control and craving were present in 97.53%, 80.24% and 79% of our study sample, respectively. In both age-wise as well as order wise chronologies, craving, tolerance and loss of control emerged as the most frequently occurring criteria during the predependence stage of alcohol dependence. Therefore, we believe that, if these criteria are routinely enquired by all clinicians for all alcohol users, especially the ‘alcoholic diagnostic orphans’, it may enable early detection and possible prevention of development of ADS. Moreover, a study(18) on one and three years follow-up of predependence alcoholic users, found a 12–13% risk of development of dependence after the appearance of any predependence criteria.

Another area of focus is the use of anticraving medications in alcohol diagnostic orphans [as a prophylactic for prevention of ADS rather than as merely relapse prevention in ADS]. Such an approach may be attempted when craving develops as the first or second criterion, even if the complete syndrome of ADS is not yet developed. It can be argued that since craving is a specific symptom in ADS, which has neurobiological underpinnings involving functional changes within neurotransmitters and receptors of the brain reward center, psychopharmacological agents have a role to play.(19) In the present study, craving was the first criterion 16% of the subjects, second criterion in 16% and third criterion in 19.8%. This represents a sizable bulk of future ADS patients, who may benefit from prophylactic anticraving medications even before the development of the clinical syndrome of alcohol dependence, as we understand it today. Active community-based, nonpharmacological methods, including behavioral interventions may possibly be useful in this population of diagnostic orphans. Such interventions have the potential to save significant resources and reduce social and economic burden to the individual as well as to the society at large. Moreover, there is evidence to suggest that brief interventions are beneficial to the ‘at-risk’ patients and problem drinkers who have not yet become alcohol dependent.(7)

The role of anticraving medications is even stronger in patients with a genetic load of ADS.(20) In our study, nearly four-fifths of the population had a positive family history of ADS. Hereditary factors have been predictive of lower ages of onset and faster progression to dependence.(21) An altered behavioral response to alcohol,(22) lower subjective response to alcohol(23) and a four-fold increase in the risk of future dependence within 10 years(24) has also been noted. Naltrexone — a prototypal anticraving agent(22) — has been shown to cause significant reduction in craving among volunteers with family history of alcoholism.(25) Alcoholic patients who drink during naltrexone treatment report less alcohol ‘high’ and are less likely to progress to heavy drinking.(26) It is possible that many of our patients with genetic load would have benefited with the use of anticraving agents before the development of dependence, ultimately preventing ADS. Since most of the alcohol dependent subjects began drinking at adolescence, which is a tendency in India as well as the rest of the world,(15) we believe that targeting adolescents with problem alcohol use may help prevent an entire generation of ‘addicts’.

***Strengths:*** The order-wise chronology along with age-wise chronology of each criterion of dependence is the uniqueness of this study. To the best of our knowledge, this is the first study conducted on chronologies of the dependence criteria. The prevalence of each criterion in our study is more or less consistent with that reported in other studies. This is the first study to discuss a strategy for primary prevention of ADS rather than only alcohol use, which is more realistic in reducing the incidence of ADS in the society at large. Even though this is a retrospective study, care has been taken to minimize the inevitable recall bias, by using more reliable and valid instruments such as SSAGA-II, MMSE screening before interview, corroboration from the CRF as well as individually framed questions in the interview.

***Limitations:*** There are inherent limitations in this study design such as inclusion of only male patients, historical cohort study recall bias even though it is reduced to a great extent and lack of generalizability (in terms of ages of onset) to a general population. However, our study presents a broad rather than a specific and detailed framework for the prevention of ADS.

***Future directions:*** With the help of a broad framework derived in our study, we suggest that studies in different target populations be carried out to formulate respective plans for primary prevention of ADS, since our findings may not be applicable to other cultures in terms of ages of onset of different criteria because of cultural variations in the criteria such as salience and tolerance. Prospective studies of the preventive interventions of the predependence alcohol users may also be carried out to determine the degree of reduction in the incidence of ADS.

## Conclusions

The chronology of dependence criteria provides us an opportunity for intervention as a step toward prevention of ADS. Routine analysis of the presence of craving, tolerance, withdrawal symptoms and loss of control in every alcohol user presenting to health care facilities would be beneficial for the prevention of ADS. Prospective studies are required to test the effectiveness of the suggested interventions in alcohol users.
